# Cellular antioxidative, cytotoxic, and antileishmanial activities of *Homalium letestui*

**Published:** 2013

**Authors:** Jude Efiom Okokon, Ahsana Dar Farooq, Mohammed Iqbal Choudhary

**Affiliations:** 1 *Department Of Pharmacology and Toxicology, Faculty of Pharmacy, University of Uyo, Uyo, Nigeria *; 2 *International Center for Chemical and Biological Sciences, University Of Karachi, Karachi, Pakistan*

**Keywords:** Antileishmanial, Antioxidant, Cytotoxic, * Homalium letestui*, Immunomodulatory

## Abstract

**Objective:** *Homalium letestui *Pellegr (Flacourtiaceae) is used in traditional medicine in parts of Nigeria for the treatment of malaria, ulcer, and inflammatory diseases and as an aphrodisiac. This investigation was aimed to evaluate the cytotoxic, immunomodulatory, and antileishmanial properties of stem extract and fractions of *Homalium letestui (H. letestui*).

**Materials and Methods:** Cytotoxic activity against HeLa cells was done using sulphorhodamine (SRB) method and DNA interaction activity using gel electrophoresis. Immunomodulatory activity of the extract in whole blood, neutrophils, and macrophages was also investigated using luminol/lucigenin-based chemiluminescence assay. The extract and fractions were similarly screened for antileishmanial activity against promastigotes of *Leishmania major *in vitro. The GCMS analysis of the most active fraction against HeLa cells was carried out.

**Results:** The stem extract exerted prominent cytotoxic activity with the dichloromethane fraction exhibiting the most pronounced effect (GI_50 _-5.12±1.45 µg/ml, LC_50_- 57.3±2.33 µg/ml, TGI -12.6±0.87 µg/ml). The crude extract and the fractions did not interact with DNA when investigated using electrophoresis. The extract significantly ((p<0.05 – 0.001) inhibited oxidative burst activity in whole blood (–27.90-66.90%), isolated polymorphonuclear cells (PMNs) (16.50-67.0%), and mononuclear cells (MNCs) (4.31-98.50%) when two different phagocytosis activators (serum opsonizing zymosan-A and PMA) were used. The extract also exhibited moderate antileishmanial activity against promastigotes of *Leishmania major *in vitro. GCMS analysis of active fraction revealed pharmacologically active compounds.

**Conclusion: **These results suggest that the stem extract/fractions of *H. letestui* possess cytotoxic, immunomodulatory, and antileishmanial activities.

## Introduction

*Homalium letestui *Pellegr (Flacourtiaceae) is a forest tree growing up to 25-30 meters and found in the rainforest of West Africa (Hutchinson & Daziel, 1963 ▶; Keay, 1989 ▶). The plant parts, particularly stem bark and root, are used in various decoctions traditionally by the Ibibios of the Niger Delta of Nigeria to treat stomach ulcer, malaria, and other inflammatory diseases as well as an aphrodisiac (Okokon et al., 2006 ▶). Reports of antiplasmodial (Okokon et al., 2006 ▶) and antidiabetic (Okokon et al., 2007 ▶) activities of the plant have been published. However, other members of the family (flacourtiaceae) and genus (*Homalium*) have been reported to possess various biological activities; anticancer activity has been reported on *Casearia capitella* (Ismail et al., 2012 ▶), *Flacourtia indica* has antioxidant activity (Madan et al., 2009; Tyagi et al., 2010 ▶), while *Homalium deplanchi *has antileishmanial, antitrypanosomal and antitrichomonal activities (Desrivot et al., 2007 ▶). *Homalium panayanum *has been reported to exert antibacterial activity against some Gram-positive and Gram-negative bacteria. (Chung et al., 2004 ▶) and *Homalium cochinchinensis *has antiviral activity (Ishikawa et al., 2004 ▶).* Casearia sylvestris* and *Casearia lasiophylla* reportedly have cytotoxic activity (Silva et al., 2008 ▶; Salvador et al., 2011 ▶) and *Homalium africanum* has antifilaricidal (Cho-Ngwa et al., 2010 ▶) activity. Cytotoxic, antioxidant, and antimicrobial activities were reported for Casearia grayi and Scolopia braunii *(*Mosaddik et al., 2004 ▶*)*. Moreover, anthelmintic activity has been reported for *Homalium zeylanicum* (Gnananath et al., 2012 ▶). Information on the pharmacology and phytochemistry of *H. letestui* is scarce. The present study attempts to evaluate the anticancer, antileishmanial, and immunomodulatory activities of this plant in order to provide scientific basis for its use in traditional medicine. 

## Materials and Methods

**Plants collection**

The plant material *H. letestui *(stem) was collected in a forest in Uruan area, Akwa Ibom State, Nigeria in April, 2011. The plant was identified and authenticated by Dr. Margaret Bassey of Department of Botany and Ecological Studies, University of Uyo, Uyo, Nigeria (Voucher no. FPUU. 382). 

**Extraction**

The stem was washed and shade-dried for two weeks. The dried plants’ materials were further chopped into small pieces and reduced to powder. The powdered material was macerated in 70% ethanol. The liquid filtrates were concentrated and evaporated to dryness in vacuo 40 C using rotary evaporator. The crude ethanolic extract (100 g) was further partitioned successively into 1 L each of n-Hexane, dichloromethane, ethyl acetate, and butanol to give the corresponding fractions of these solvents (Okokon et al., 2012 ▶).

**Cellular antioxidant (Immunomodulatory) activity**

The ethanolic crude extract was screened for cellular antioxidant activities in whole blood, neutrophils, and macrophages using chemiluminescence assay. Briefly, Luminol or lucigenin-enhanced chemiluminescence assay was performed as described by Helfand et al. (1982) ▶ and Haklar et al. (2001) ▶. Briefly, 25 µL diluted whole blood (1:50 dilution in sterile HBSS^++^) or 25 µL of PMNCs (1×10^6^) or MNCs (5×10^6^) cells were incubated with 25 µL of serially diluted plant extract with concentration ranges between 6.25 and 100 µg/mL. Control wells received HBSS++ and cells but no extract. Tests were performed in white 96 wells plates, which were incubated at 37 C for 30 min in the thermostated chamber of the luminometer. Opsonized zymosan-A or PMA 25 µL, followed by 25 µL luminol (7×10^5 ^M) or lucigenin (0.5 mM) along with HBSS^++^ was added to each well to obtain a 200 µL volume/well. The luminometer results were monitored as chemiluminescence RLU with peak and total integral values set with repeated scans at 30 s intervals and 1 s points measuring time.

**Cytotoxic activity**

The growth inhibitory and cytotoxic activities of the ethanolic extract and fractions were evaluated against HeLa cells (Cervix cancer cell) by using the sulforhodamine-B assay (Houghton et al., 2007 ▶). The cells (10000 cells/100 µL) in 96-well plate were incubated for 24 h at 37 °C in a humidified 5% CO_2_ incubator. The stock solutions of ethanolic extract, fractions were prepared in DMSO. Various dilutions of the ethanolic extracts and fractions (0.1, 1, 10, 100, and 250 µg/mL), were added (100 µL) in each well. After 48 h of incubation, 50 µL of cold TCA (50%) was added gently and left for 30 min at room temperature, followed by washing with distilled water and drying overnight. To each well, 100 µL of SRB solution (0.4% wt/vol in 1% acetic acid) was added and after 10 min, the unbound stain was removed by washing with acetic acid (1%), and air-dried at room temperature. The protein bound stain was solubilized with tris base (pH 10.2), and was shaken for 5 min. Absorbance was measured at 515 nm using a microplate reader. The absorbance of the appropriate blanks, including test substance blank, and control (without drug) was used to calculate the growth inhibition and cytotoxicity of the test compounds and represented as GI_50_, TGI, and LC_50_ (µg/mL) values.

**DNA interaction study using gel electrophoresis**

DNA interaction assay was performed according to the protocol of Tian and Hua (2005) ▶. The reaction was carried out in an Eppendorf tube at the total volume of 15 μl containing 0.5 μg of pBR322 DNA in 3 μl of 50 mM phosphate buffer (pH 7.4), and 5 μl of tested samples (DCM fraction) at concentrations 0.1, 0.5, 1.0, 10, 50, and 100 μg/ml and standard drug, paclitaxel, 20 µg/mL. Then, the mixture was incubated at 37 °C for 1 h. The mixture was subjected to 1% agarose gel electrophoresis. DNA bands (open circular, supercoiled, and linear) were stained with ethidium bromide and were analyzed qualitatively by scanning with Doc-IT computer program (VWR). 

**Antileishmanial activity**

The antileishmanial activity of the extracts and fractions were evaluated against promastigotes of *Leishmania major* (DESTO) in culture using microplates. *Leishmania major (L. major*) promastigotes were grown in bulk, early in modified NNN biphasic medium, using normal physiological saline. Then, the promastigotes were cultured with RPMI 1640 medium supplemented with 10% heat inactivated fetal bovine serum (FBS). The parasites *L. major *were harvested at log phase and centrifuged at 3000 rpm for 10 min. 

They were washed three times with saline at same speed and time. Finally, the parasites were counted with the help of Neubauer chamber under the microscope and diluted with fresh culture medium to give a final density of 10^6 ^cells/ml. In a 96-well microtiter plate, 180 ml of the culture medium was added in different wells. The extracts and fractions were dissolved in PBS (Phospate buffered saline, pH 7.4 containing 0.5 % MeOH, 0.5 % DMSO) to make a stock concentration of 1000 mg/ml. Twenty µl of each extract/fraction concentration was added to the wells and serially diluted to get working concentrations ranging between 1.0 to 100 µg/ml. One-hundred ml of parasite culture (final density of 10^6 ^cells/ml) was added in all wells. Two rows were left, one for negative and other for positive control. Negative controls received the medium while the positive controls received pentamidine and amphotericin B as standard antileishmanial compounds. The plate was incubated between 21-22 °C for 72 h. The culture was examined microscopically for cell viability by counting the number of motile cells on an improved Neubauer counting chamber and IC_50 _values of compounds possessing antileishmanial activity were calculated (Atta-ur-Rahman, 2001 ▶).

**GC-MS analysis of fractions**

Quantitative and qualitative data were determined by GC and GC-MS, respectively. The fraction was injected onto a Shimadzu GC-17A system, equipped with an AOC-20i autosampler and a split/splitless injector. The column used was an DB-5 (Optima-5), 30 m, 0.25 mm i.d., 0.25 µm df, coated with 5% diphenyl-95% polydimethylsiloxane, operated with the following oven temperature programme: 50 °C, held for 1 min, rising at 3 °C/min to 250 °C, held for 5 min, rising at 2 °C/min to 280 °C, held for 3 min; injection temperature and volume, 250 °C and 1.0 µl, respectively; injection mode, split; split ratio, 30:1; carrier gas, nitrogen at 30 cm/s linear velocity and inlet pressure 99.8 KPa; detector temperature, 280 °C; hydrogen, flow rate, 50 ml/min; air flow rate, 400 ml/min; make-up (H2/air), flow rate, 50 ml/min; sampling rate, 40 ms. Data were acquired by means of GC solution software (Shimadzu).

Agilent 6890N GC was interfaced with a VG Analytical 70-250s double focusing mass spectrometer. Helium was used as the carrier gas. The MS operating conditions were: ionization voltage 70 eV, ion source 250 °C. The GC was fitted with a 30 m×0.32 mm fused capillary silica column coated with DB-5. The GC operating parameters were identical with those of GC analysis described above.

The identification of components present in the various active fractions of the plants’ extracts was based on direct comparison of the retention times and mass spectral data with those for standard compounds, and by computer matching with the Wiley and Nist Library, as well as by comparison of the fragmentation patterns of the mass spectra with those reported in the literatures (Adams, 2001 ▶; Setzer et al., 2007 ▶).

**Statistical analysis**

Data are reported as mean±SEM and were analyzed statistically using Tukey-kramer multiple comparison test and values of p<0.001 and 0.05 were considered significant.

## Results

The results of cytotoxic activity of crude extract and fractions of *Homalium letestui* show prominent activity with the hexane fraction exerting highest activity than other fractions and crude extract (Table 1). The potency order was dichloromethane > ethylacetate > butanol > hexane > aqueous > crude extract.

**Cytotoxic activity against HeLa cells**

*Gel Electrophoresis*

**Figure 1:**
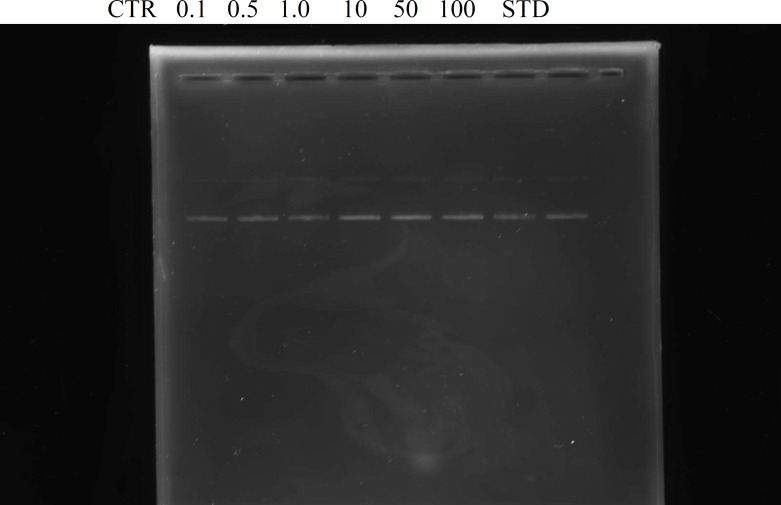
The effect of various concentrations of hexane fraction of *H. letestui* on DNA interaction using gel electrophoresis

Gel electrophoresis results show that treatment of *E. coli* DNA with various concentrations of the hexane fraction of *H. letestui* did not produce any effect on the DNA. This effect was also observed with the standard drug used, paclitaxel (Figure 1). 

**Cellular antioxidant activity**

Ethanolic stem extract of *H. letestui *was observed to produce significant (p<0.05 – 0.001) inhibitory effect on the oxidative burst activities of neutrophils and macrophages activated with Zymosan-A or PMA in a dose-dependent manner especially at the highest doses used. In the whole blood, the extract exhibited pro-oxidant activity at low doses (1-10 µg/ml) and antioxidant activity at the highest dose (100 µg/ml) with inhibitory effect of 59.6% (Table 2). 

**Table 1:** Cytotoxic activity of crude extract and fractions of stem of *Homalium letestui *against HeLa cells

**Extract/Fraction**	**GI**_50 _(µg/ml)	**LC**_50 _(µg/ml)	**TGI **(µg/ml)
**Crude Extract**	116.0±1.45	240.3±3.46	204.6±1.45
**Hexane Fraction**	60.0±0.87	-	77.0±1.52
**DCM Fraction**	5.12±1.45	57.3±2.33	12.6±0.87
**Ethyl Acetate Fraction**	4.33±0.87	60.3±2.60	11.0±1.52
**Butanol**	16.0±2.31	68.2±4.50	9.66±1.20
**Aqueous Fraction **	64.6±0.87	-	81.3±1.66
**Doxorubucin**	0.61±0.03 µM	7.80±0.80 µM	3.60±0.30 µM

Data are represented as mean±SEM of three independent experiments. Values in the table are concentrations of extract/fraction expressed as µg/ml, GI_50_=Concentration of the drug causing 50% growth inhibition of the cells, TGI = Concentration of the drug causing total growth inhibition of the cells, LC_50_ = Lethal concentration of the drug that killed 50% of the cells.

**Table 2:** Cellular antioxidant activity of ethanolic stem extract of *Homalium letestui*

**Cell type**	**Dose (µg/Ml)**	**%Inhibition (Rlu)**
**Whole Blood**	1	-7.80±2.77
	10	-2.70±0.82
	100	59.6 ±1.44^b^
**Neutrophils (Intracellular)**	0.5	0.00±0.00
	5	13.50 ±3.87^a^
	50	70.20 ± 3.52^c^
**Neutrophils (Extracellular)**	0.5	30.40± 1.79^a^
	5	51.10± 2.60^b^
	50	65.70 ± 7.16^c^
**Macrophages**	0.5	31.30±4.39^a^
	5	56.60±3.58^b^
	50	73.10±2.19^c^

Data are represented as mean±SEM of three independent experiments. Significant at:

a p< 0.05,

b p< 0.01,

c p< 0.001 when compared to control.

**Antileishmanial activity**

Crude extract and fractions of ethanolic stem extract of *H. letestui *exerted significant antileishmanial activity when tested against promastigotes of *L. major*. Ethyl acetate fraction exerted a higher activity than other fractions and crude extract though uncomparable to the standard drugs, pentamidine, and amphotericin B (Table 3).

**GC-MS analysis**

The results of GCMS analysis of dichloromethane fraction of stem extract of *H. letestui *revealed the presence of pharmacologically active compounds as shown on Table 4.

**Table 3:** Antileishmanial activity of *Homalium letestui* (ED_50_).

**Extract/Fraction**	**ED**_50_(µg/ml)
**Crude Extract**	26.90±0.56
**Hexane Fraction**	>100
**DCM Fraction**	63.06±1.90
**Ethyl Acetate Fraction**	21.50±0.16
**Butanol Fraction**	>100
**Aqueous Fraction **	>100
**Pentamidine**	5.09±0.04
**Amphotericin B**	0.29±0.05

Data are represented as mean±SEM (n=3).

**Table 4:** GC –MS Analysis of Dichloromethane Fraction of *Homalium letestui*

**S/No.**	**Name of Compound **	**Mol.Wt**	**Chemical Formula**	**RI**	**Concentration %**
1.	**2,4 Heptadien-6-ynal,(E,E) **	106	C_7_H_6_O	25	0.912
2.	**2-(4-formyphenyloxy)-acetic acid**	180	C_9_H_8_O_4_	172	0.206
3.	**2-Coumaranone**	134	C_8_H_6_O_2_	195	0.968
4.	**Benzoic acid**	122	C_7_H_6_O_2_	200	0.202
5.	**4-Hepten-3-one,5-methyl,(E)-**	126	C_8_H_14_O_2_	207	8.876
6.	**Salicyl alcohol**	124	C_7_H_8_O_2_	234	3.286
7.	**4-Hepten-3-one,5-methyl,(E)-**	126	C_8_H_14_O	207	0.832
8.	**Vanillin**	152	C_8_H_8_O_3_	312	0.915
9.	**3,4,5-trimethoxy phenol**	184	C_9_H_12_O_4_	456	5.761
10.	**2,4 Decadienal,(E,Z)**	152	C_10_H_16_O	428	0.872
11.	**4-(3-hydroxy-1-propenyl)-2-methoxy phenol**	180	C_10_H_12_O_3_	527	2.017
12.	**4-hydroxy-3,5-dimethoxy Benzaldehyde**	182	C_9_H_10_O_4_	477	0.866
13.	**5,6-dimethoxyphthaldehydic acid**	210	C_10_H_10_O_5_	662	0.634
14.	**Benzene(methylsulfinyl) methyl**	154	C_8_H_10_O_5_	596	0.595
15.	**4-phenyl isocoumarin**	222	C_15_H_10_O_2_	697	2.147
16.	**2,4-dinitrophenyl hydrazine butanal**	252	C_12_H_12_N_4_O_4_	743	0.281
17.	**9H-Xanthen-9-one, 1,3-dihyroxy-4-methyl**	242	C_14_H_10_O_4_	789	1.580
18.	**Methanone,(2,4-dihydroxyphenyl) phenyl**	214	C_13_H_10_O_3_	805	2.147
19.	**1,2,3,4-tetrahydro-5,8-dimethoxy-9,10-anthracenedione**	272	C_16_H_16_O_4_	950	1.491
20.	**Camphor**	327	C_19_H_21_NO_4_	1153	5.589
21.	**α-Terpineol**	154	C_10_H_18_O	1185	15.642

## Discussion

*Homalium letestui *is used traditionally in the treatment of various ailments and diseases. The stem which has been reported to possess some pharmacological properties have been found in this study to exert pronounced cytotoxic activity against HeLa cells with the dichloromethane fraction having the highest activity. The cytotoxic mechanism of action was found to be unrelated to DNA interaction and is likely to involve interference with cell division processes. Anticancer and cytotoxic activities against cancer cell lines have been reported on *Casearia sylvestri*s (Silva et al., 2008 ▶), *Casearia lasiophylla* (Salvador et al., 2011 ▶), *Flacourtia indica* (Pachute et al., 2011 ▶), and *Casearia capitella* (Ismail et al., 2012 ▶), all members of flacourtiaceae family and the activity has been ascribed to the presence of monoterpenes and sesquiterpenes in these plants as well as the presence of phenolic and polyphenolic compounds. However, the GCMS analysis revealed the presence of some pharmacologically active compounds such as anthracenedione, 2,4-Decadienal, (E,Z), vanillin, and 1,3-dihydroxy-4-methyl-9H-Xanthen-9-one which have been implicated in the anticancer activity of plants (Nappez et al.,1996 ▶; Murakami et al., 2007 ▶; Pedraza-Chaverri et al., 2008 ▶; Zhang et al. 2010 ▶; Mansour et al., 2010 ▶). These compounds and others are likely to be involved in the cytotoxic activity of this extract.

The stem extract was also observed to exhibit strong antioxidant activity in whole blood, neutrophils (extracellular and intracellular), and macrophages. This activity may have resulted from the presence of some polyphenolic compounds such as vanillin, 2-Coumaranone, 3, 4, 5-trimethoxy phenol and 4-phenyl isocoumarin, and 4-(3-hydroxy-1-propenyl)-2-methoxy phenol as well as monoterpene (α-Terpineol) as revealed by GCMS analysis. These compounds have been reported to possess antioxidant activity (Murakami et al., 2007 ▶; Raja et al., 2011 ▶; Soobrattee et al., 2005 ▶; Falah et al., 2008 ▶). Other members of the family such as *Homalium brachybotrys, Flacourtia indica*, Casearia grayi, and Scolopia braunii have been reported to possess antioxidant activities due to presence of polyphenolic compounds (Tyagi et al., 2010 ▶; Mosaddik et al., 2007 ▶; Mosaddik et al., 2004 ▶*)*. The GCMS analysis also revealed the presence of some phenolic compounds such as xanthones which have been implicated for many biological activities such as antioxidant, antitumoral, anti-inflammatory, antiallergy, antibacterial, antifungal, and antiviral activities (Pedraza-Chaverri et al., 2008 ▶; Suksamrarn et al., 2006 ▶). These compounds present in this plant’s extract may be responsible for its antioxidant activity reported in the current study. The extract as well as the fractions especially dichloromethane fraction possess a significant cytotoxic activity against HeLa cells in culture. The significant antioxidant activity of this extract explains the strong cytotoxic activity of the stem extract. Generation of reactive oxygen species has been implicated in the pathogenesis of cancer and other diseases (Halliwel and Gutteridge, 1999 ▶). The activities of antioxidant counteract the redox state precipitated intracellularly and hence ensure cytotoxicity. This could possibly be one of the mechanisms of cytotoxic activity of this extract.

The stem extract also demonstrated antileishmanial activity. The extract was also observed to possess antileishmanial activity on *L. major*. *Homalium deplanchi *has also been reported to have antileishmanial, antitrypanosomal, and antitrichomonal activities (Desrivot et al., 2007 ▶) indicating a strong antiprotozoal activity. Antimicrobial activities are known to be promoted by proxidant state. In this study, lower doses of the extract have been observed to exhibit pro-oxidant activity. This activity has been reported to enhance antimicrobial activity (Anderson et al., 1981 ▶). Moreover, bioactive compounds such as xanthones which have been implicated in immune stimulation and antimicrobial activities have been reported to be present in this extract. Xanthones have been reported to possess antileishmanial activity (Mbwambo et al., 2006 ▶) and camphor, a well-known chemical with its pronounced antimicrobial potentials (Pattnaik et al., 1997 ▶) is also present in this extract. These compounds present in this plant may be responsible for its antileishmanial activity. This is the first report of antileishmanial activity of *H. letestui*. 

From the results of this study, it can be concluded that the stem bark extract of *H. letestui *has cytotoxic activity against HeLa cells, immunomodulatory and antileishmanial activities which are due to the phytochemical constituents of the extract and fractions.
